# Repeated episodes of postictal hypoxia are a mechanism for interictal cognitive impairments

**DOI:** 10.1038/s41598-023-42741-7

**Published:** 2023-09-19

**Authors:** Bianca R. Villa, Dhyey Bhatt, Marshal D. Wolff, Kwaku Addo-Osafo, Jonathan R. Epp, G. Campbell Teskey

**Affiliations:** 1 Cumming School of Medicine, Hotchkiss Brain Institute, University of Calgary, Calgary, AB T2N 4N1 Canada; 2 Department of Cell Biology and Anatomy, University of Calgary, 3330 Hospital Drive NW, Calgary, AB T2N 4N1 Canada; 3 Cumming School of Medicine, Alberta Children’s Hospital Research Institute, University of Calgary, Calgary, AB T2N 4N1 Canada

**Keywords:** Epilepsy, Molecular neuroscience

## Abstract

Comorbidities during the period between seizures present a significant challenge for individuals with epilepsy. Despite their clinical relevance, the pathophysiology of the interictal symptomatology is largely unknown. Postictal severe hypoxia (PIH) in those brain regions participating in the seizure has been indicated as a mechanism underlying several negative postictal manifestations. It is unknown how repeated episodes of PIH affect interictal symptoms in epilepsy. Using a rat model, we observed that repeated seizures consistently induced episodes of PIH that become increasingly severe with each seizure occurrence. Additionally, recurrent seizure activity led to decreased levels of oxygen in the hippocampus during the interictal period. However, these reductions were prevented when we repeatedly blocked PIH using either the COX-inhibitor acetaminophen or the L-type calcium channel antagonist nifedipine. Moreover, we found that interictal cognitive deficits caused by seizures were completely alleviated by repeated attenuation of PIH events. Lastly, mitochondrial dysfunction may contribute to the observed pathological outcomes during the interictal period. These findings provide evidence that seizure-induced hypoxia may play a crucial role in several aspects of epilepsy. Consequently, developing and implementing treatments that specifically target and prevent PIH could potentially offer significant benefits for individuals with refractory epilepsy.

## Introduction

Epilepsy is a common neurological disease, often complicated by interictal (the time between seizure) behavioural comorbidities^1^. Cognitive abnormalities are among the most common and severe, and include memory impairment, learning disabilities as well as a broader pattern of cognitive decline in areas such as IQ, executive function, and language skills^2–4^. The presence of such comorbidities significantly diminishes the quality of life for individuals with epilepsy. Despite their clinical relevance, the pathophysiology is largely unknown. It is crucial to investigate and determine the mechanisms responsible for seizure-induced brain dysfunction and to develop effective therapeutic interventions to address this unmet medical need.

In 2016, Farrell et al. identified extended hypoperfusion/severe hypoxia rather than the seizures themselves, as a mechanism underlying acute postictal cognitive and behavioural deficits following temporal lobe seizures^5^. The postictal hypoxia (PIH) phenomenon has been determined to be partly mediated by arteriole vasoconstriction with enzyme cyclooxygenase-2 (COX-2) and L-type calcium channels playing important mechanistic roles^5^. During seizure activity, the induction of neuronal COX-2 leads to oxygenation of arachidonic acid, resulting in the formation of vasoactive products. These products then act on receptors located on vascular smooth muscle, causing the opening of L-type calcium channels, and leading to long-lasting vasoconstriction^5^. Additionally, there is evidence indicating that mitochondrial dysfunction and production of reactive oxygen species (ROS) also contributes to both postictal hypoxia (PIH) and postictal cognitive impairment^6^.

To investigate the potential causal relationship between episodes of PIH and interictal behavioural dysfunction, we employed the electrical kindling model of seizures^7^. In support, several studies have reported the development of long-term cognitive deficits following even minor transient ischemic/hypoxic attacks^8–10^. Our findings revealed a consistent pattern of repeated PIH events in the hippocampus of kindled rats, which increased in severity with each subsequent seizure. These repeated episodes of PIH led to decreased interictal oxygen levels in the hippocampus and resulted in long-lasting memory deficits, while having no impact on the progression of kindling (epileptogenesis). Mitochondrial dysfunction may also contribute to the observed pathological outcomes during the interictal period. Collectively, these results bring new insights to the mechanistic basis of interictal symptomatology and its relationship to postictal hypoxia. They also established PIH as a promising target for therapeutics aimed at addressing various negative aspects of epilepsy, which is especially relevant for individuals who have poor seizure control and are unresponsive to existing medications.

## Results

### Seizure-induced hypoxic events become progressively more severe, and this is prevented by both acetaminophen and nifedipine

**Figure 1:**
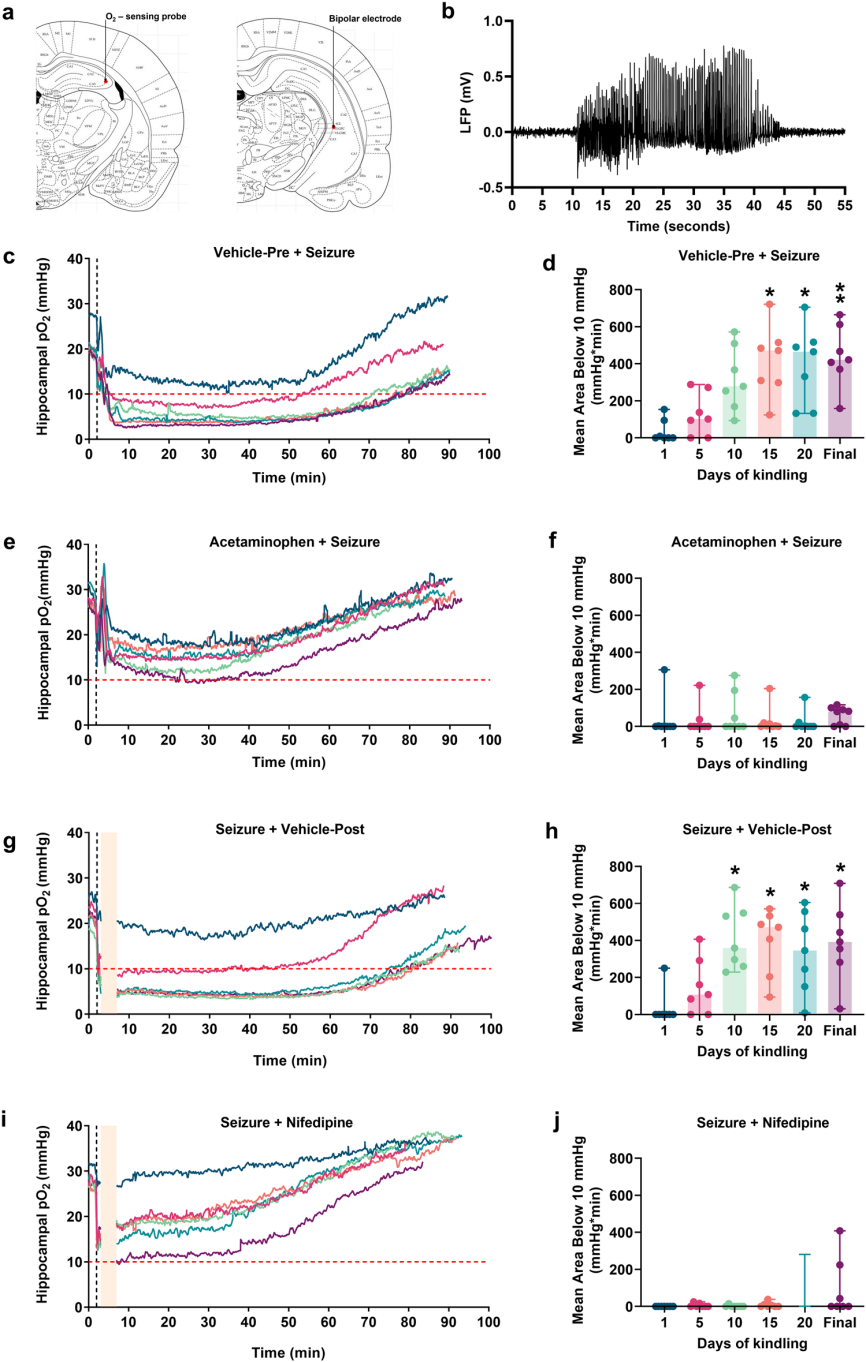
Repeated seizures induce repeated postictal hypoxic (PIH) events. Rats were injected with either acetaminophen (250 mg/kg; 30 min prior to each kindling session) or nifedipine (15 mg/kg; immediately following seizure cessation) or their vehicles for 20 days. (**a**) Brain atlas plate depicting location of oxygen-sensing probe (left) and electrode stimulation site (right) in the CA3 area of the dorsal and ventral right hippocampus, respectively. (**b**) Representative EEG tracing depicting spike activity occurring during electrically induced seizures. *LFP* local field potential. (**c**,**e**,**g**,**i**) Hippocampal oxygen traces depicting changes in local pO_2_ before, during, and after electrically induced seizures. Different coloured traces indicate different kindling day. Horizontal dashed red line indicates the severe hypoxic threshold of 10 mmHg. Vertical dashed black line indicates seizure stimulation. Vertical light-yellow bar indicates when rats were unplugged from oxygen measurements to receive injections. Mean ± SEM (n = 7–8 each group). (**d**,**f**,**h**,**j**) Quantification of degree of severe hypoxia expressed as the area spent under the severe hypoxic threshold (10.0 mmHg) by time (min) for each experimental group (n = 7–8 each group). The Shapiro–Wilk test indicated a significant departure from normality (**p < 0.01) in the data distribution. Data are shown as median (95% CI), followed by non-parametric Friedman repeated measure ANOVA and Dunn’s post hoc test (*p < 0.05; **p < 0.01 versus Day 1 of kindling).

**Figure 2:**
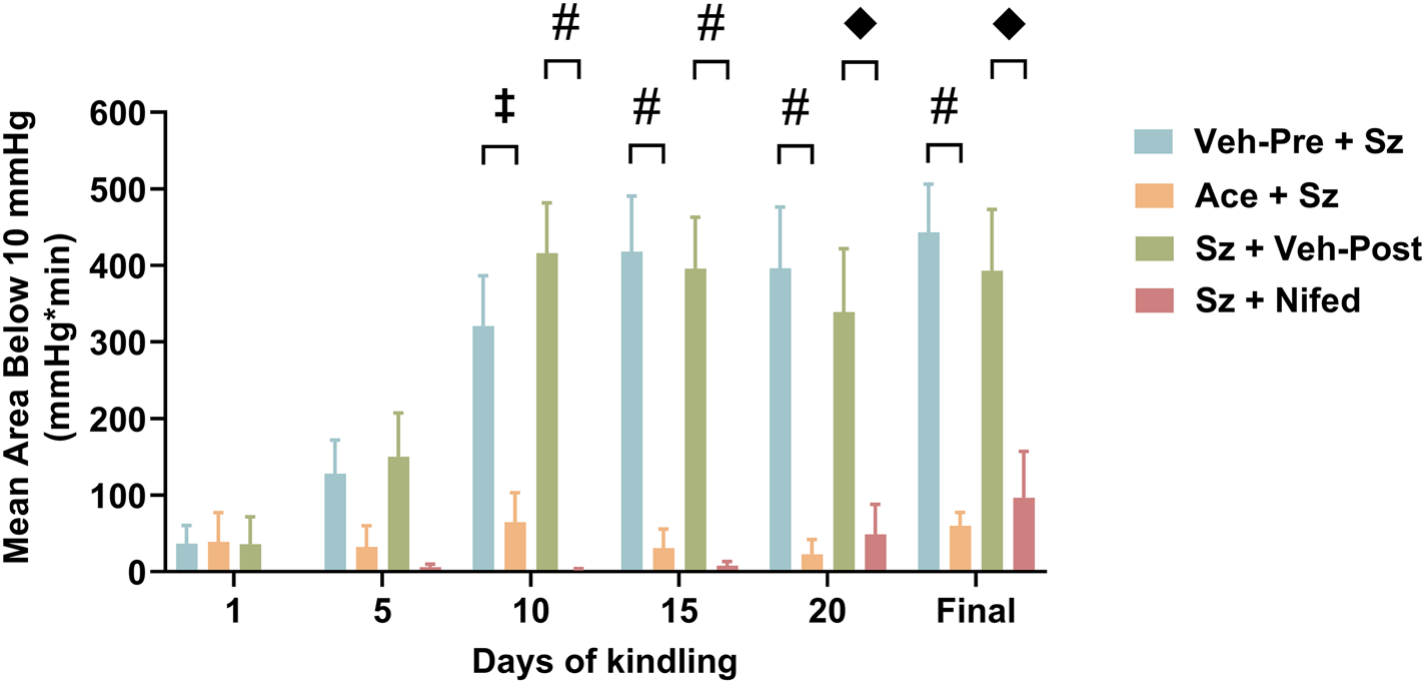
Acetaminophen and nifedipine treatment decrease PIH severity during kindling. Severity of PIH during kindling for the different treatment groups expressed as the mean area spent under the severe hypoxic threshold (10.0 mmHg) by time (min; n = 7–8 each group). Data are shown as mean ± SEM, followed by two-way ANOVA and Tukey’s post hoc test (^‡^p < 0.01; ^♦^p < 0.001; ^#^p < 0.0001). *Veh* vehicle, *Sz* seizure, *Ace* acetaminophen, *Nifed* nifedipine.

We tracked the progression of the severity of PIH in the rat hippocampus over the course of electrical kindling (Fig. 1). Rats were chronically implanted with an oxygen-sensing probe into the dorsal right hippocampus and an electrode in the ventral right hippocampus to elicit hippocampal afterdischarges (Fig. 1a,b). Severe hypoxic profiles were quantified by integrating duration and depth of oxygen levels below the critical threshold (10 mmHg), known for causing brain injury and adverse clinical outcomes^11^. Thirty minutes prior to each kindling session, rats were injected with the COX-inhibitor acetaminophen, which has been previously shown to acutely block PIH when administered prior to seizures, or its vehicle (Fig. 1c–f)^5, 12^. Vehicle-treated rats showed a progressive increase in PIH severity over time (Q = 17.5, p = 0.004; Fig. 1c,d). Specifically, there were statistically significant increases in the AB10 on days 15 (417.8 ± 72.61 mmHg min), 20 (396.4 ± 79.79 mmHg min) and the final kindling session (443.3 ± 62.89 mmHg min) compared to days 1 (36.83 ± 23.48 mmHg min). Conversely, the severity of PIH did not change throughout kindling for rats receiving acetaminophen (Q = 7.6, p = 0.18; Fig. 1e,f). A separate cohort of kindled rats were treated with nifedipine—an L-type calcium channels antagonist that has proven effective at blocking PIH when given after seizures—or its vehicle immediately following seizure cessation (Fig. 1g–j)^5, 12^. Similarly, PIH reached maximal severity on days 10 (Q = 18, p = 0.003; 415.9 ± 65.55 mmHg min) compared to days 1 (35.77 ± 35.77 mmHg min) before plateauing for rats receiving vehicle post seizures (Fig. 1g,h). Nifedipine prevented the worsening of PIH severity over time (Q = 7.89, p = 0.16; Fig. 1i,j). Finally, the severity of PIH was compared among groups (Fig. 2). Vehicle-treated groups had significantly higher AB10 than both acetaminophen and nifedipine-treated groups (F_3,25_ = 24.61, p < 0.0001; Fig. 2) on days 10, 15, 20, and the final kindling day. We can conclude that repeatedly triggering seizures in the hippocampus consistently results in recurrent PIH events that gradually worsen in severity. However, administering acetaminophen or nifedipine repeatedly prevented the occurrence of PIH.

### Repeated PIH events do not affect kindling progression

**Figure 3:**
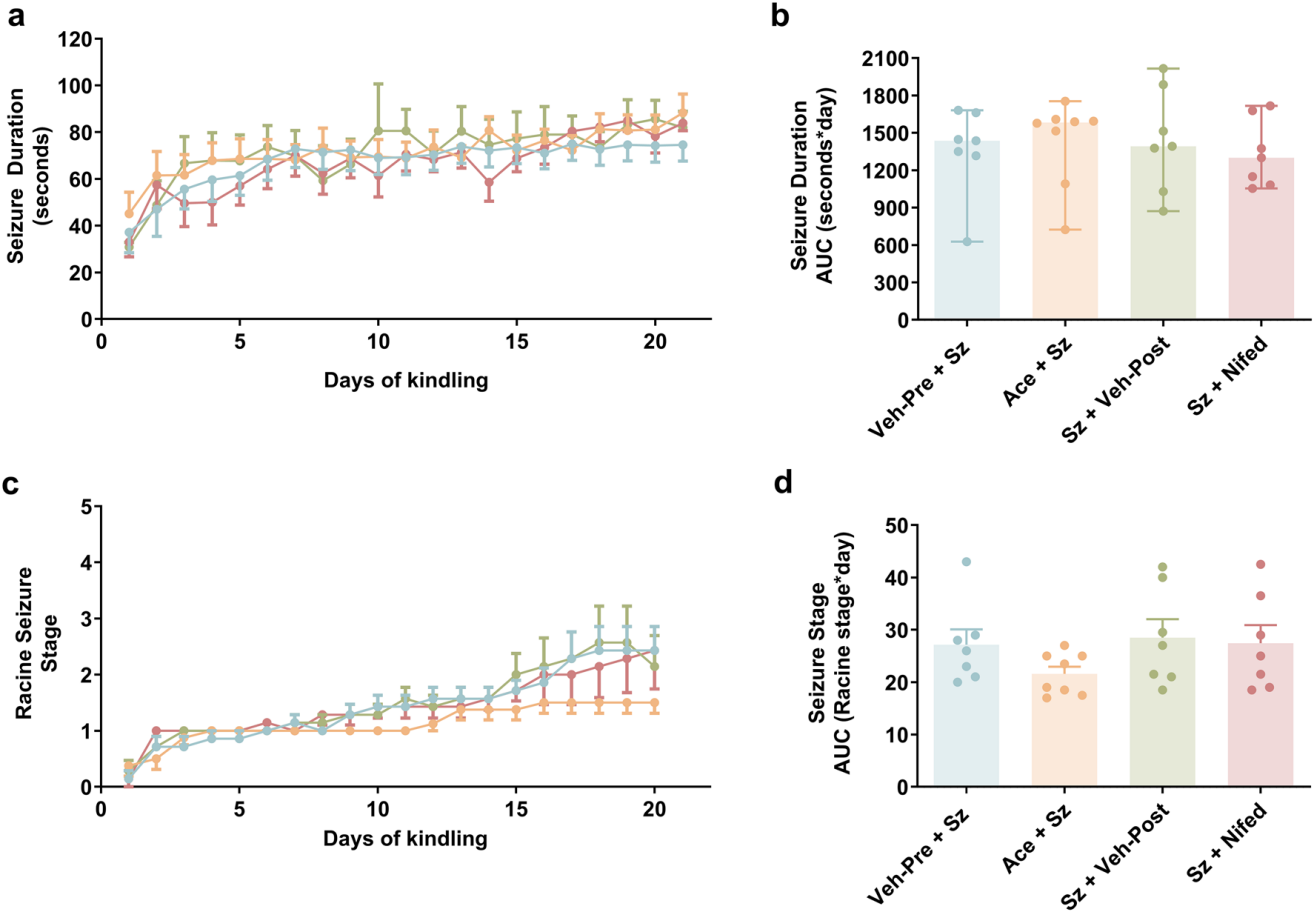
Repeated PIH events do not influence seizure duration or seizure stage. (**a**) Mean seizure duration plotted over the course of kindling. Different coloured traces indicate different treatments: blue (vehicle pre-seizure), orange (acetaminophen), green (vehicle post-seizure), and red (nifedipine). Mean ± SEM (n = 7–8 each group). (**b**) Quantification of seizure duration severity expressed as the area under the curve (AUC). The Shapiro–Wilk test indicated a significant departure from normality (**p < 0.01) in the data distribution. Data are shown as median (95% CI; n = 7–8 each group), followed by non-parametric Kruskal–Wallis ANOVA and Dunn’s post hoc test. (**c**) Mean Racine seizure stage plotted over the course of kindling. Mean ± SEM (n = 7–8 each group). (**d**) Quantification seizure stage severity expressed as the area under the curve (AUC). The Shapiro–Wilk test indicated data are normally distributed. Data are shown as mean ± SEM (n = 7–8 each group), followed by one-way ANOVA and Sidak’s post hoc test. *Veh* vehicle, *Sz* seizure, *Ace* acetaminophen, *Nifed* nifedipine.

Based on our time-course data on the severity of PIH, we further interrogated whether any progressive enhancement of seizure severity is causally related to repeated episodes of PIH. We adopted acetaminophen and nifedipine as experimental tools to dissociate the contribution of PIH from the contribution of the seizure itself. We observed seizure duration progressively increased following repeated electrical stimulation of the hippocampus (Fig. 3a). However, blocking PIH with either acetaminophen or nifedipine did not alter the progressive increase in seizure length (H = 0.93, p = 0.82; Fig. 3b). Similarly, the severity of behavioural seizures progressively increased over kindling as indicated by the significant increase in seizure stage (Fig. 3c) but blocking PIH did not affect the progressive increase in seizure stage (F_3,25_ = 1.25, p = 0.31; Fig. 3d). While the severity of PIH increases because of repeated seizures, repeated PIH does not contribute to kindling progression.

### Interictal hippocampal pO_2_ decreases with repeated seizures and can be prevented by blocking PIH

**Figure 4:**
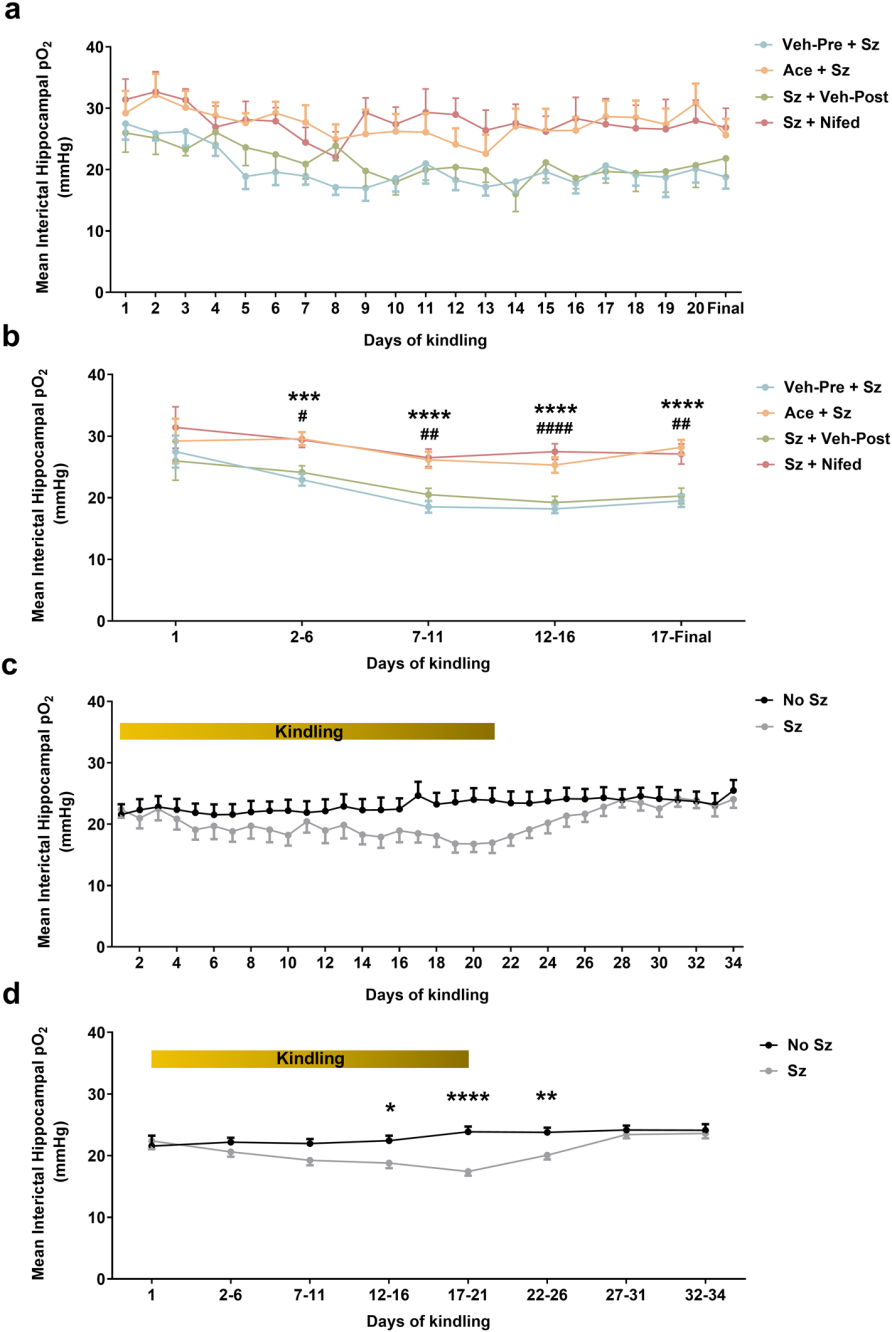
Interictal hippocampal pO_2_ decreases during kindling and this is prevented by repeated attenuation of PIH. Ten-minute interictal pO_2_ was recorded in rat hippocampus over the course of kindling and for the following two weeks. (**a**) Hippocampal oxygen traces depicting changes in local pO_2_ in between seizures for rats receiving pharmacological treatment (acetaminophen: orange; nifedipine: red) and rats receiving vehicle (pre-seizure: blue; post-seizure: green). Mean ± SEM (n = 7–8 each group). (**b**) Hippocampal oxygen traces depicting changes in local pO_2_ when kindling days were binned. Data are shown as mean ± SEM (n = 9 each group), followed by two-way repeated measure ANOVA and Sidak’s post hoc test (***p < 0.001; ****p < 0.0001 versus Veh-Pre + Sz; ^#^p < 0.05; ^##^p < 0.01; ^####^p < 0.0001 versus Sz + Veh-Post). (**c**) Hippocampal oxygen traces depicting changes in local pO_2_ between seizures for seizure exposed hypoxic rats (gray) and control rats that did not received kindling stimulation (black). Mean ± SEM (n = 9 each group). (**d**) Hippocampal oxygen traces depicting changes in local pO_2_ when kindling days were binned. Data are shown as mean ± SEM (n = 9 each group), followed by two-way repeated measure ANOVA and Sidak’s post hoc test (*p < 0.05; **p < 0.01; ****p < 0.0001 versus No Sz). *Veh* vehicle, *Sz* seizure, *Ace* acetaminophen, *Nifed* nifedipine, *No Sz* no seizure.

We then tested the potential impact of recurrent PIH episodes on interictal oxygen levels (Fig. 4). Every day over the course of the experiment, partial pressure of oxygen (pO_2_) was recorded for 10 min from the rat dorsal hippocampus 24 h after a previous seizure (Fig. 4a). There was a significant overall effect of treatment on the mean interictal baseline pO_2_ (F_3,141_ = 15.29, p < 0.0001; Fig. 4b). Specifically, vehicle-treated rats had significantly lower interictal baseline hippocampal pO_2_ compared to acetaminophen or nifedipine-treated rats starting from days 2–6 (Fig. 4b). Acetaminophen or nifedipine treatment prevented interictal oxygen levels to drop following seizures. We can conclude that repeated PIH, and not seizures themselves, lower oxygen levels between seizures. Finally, we investigated if the effect of repeated PIH on tissue oxygenation was long-lasting by recording interictal hippocampal pO_2_ for 2 weeks after the final seizure (Fig. 4c). We observed a significant effect of seizure activity on interictal oxygen levels (F_1,88_ = 5.72, p = 0.02; Fig. 4d). Specifically, rats that exhibited seizures had significantly lower interictal oxygen levels at the end of kindling that recovered back to baseline by 6 days from the final seizure (Fig. 4d).

### Repeated seizures lead to long-term interictal memory impairment that is prevented by blocking repeated PIH

**Figure 5:**
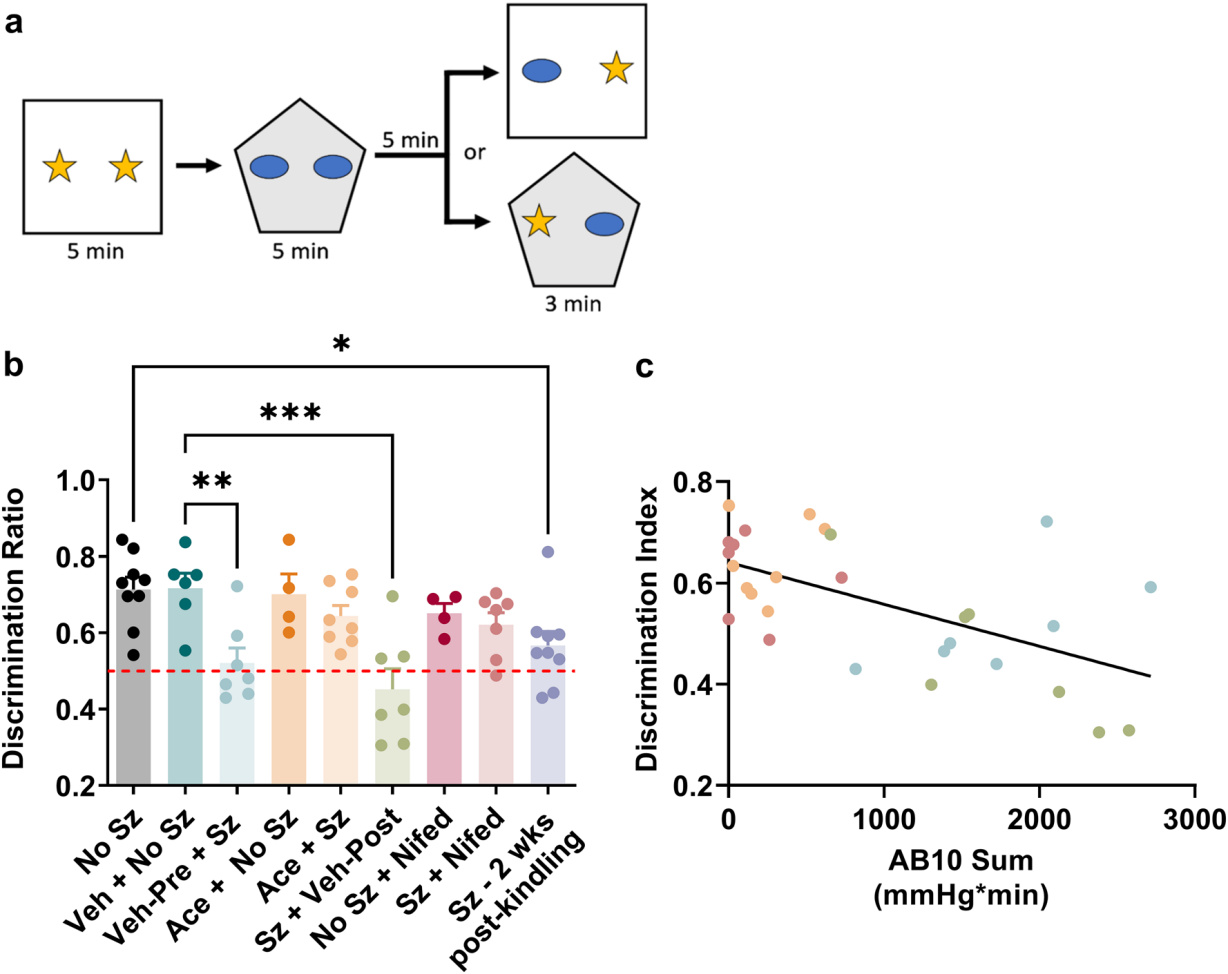
Interictal memory impairments induced by kindling are long-lasting and can be prevented by blocking repeated PIH. Hippocampal—dependent novel object/context-mismatch task was performed in rats 24 h and 2 weeks after the last seizure. (**a**) Schematic representation of novel object/context-mismatch task. (**b**) Quantification of discrimination index. The horizontal red dashed line represents a discrimination index of 0.5. Values above 0.5 indicates greater investigation of the novel object, thus preserved memory formation. The Shapiro–Wilk test indicated data are normally distributed. Data are shown as mean ± SEM (n = 4–9 each group), followed by one-way ANOVA and Sidak's post hoc test (*p < 0.05; **p < 0.01; ***p < 0.001). (**c**) Scatterplot of the relationship between the degree of severe hypoxia expressed as the sum of the total area below the severe hypoxic threshold (10.0 mmHg) by time (min) over kindling and the discrimination index. The line of best fit (Y =  − 8.306e−005 × X + 0.6411) is indicated. R^2^ = 0.35, p = 0.0007. *Veh* vehicle, *Sz* seizure, *Ace* acetaminophen, *Nifed* nifedipine, *No Sz* no seizure, *Wks* weeks.

To determine the influence of recurrent episodes of PIH on interictal behavioral disruption, we tested the rats in the novel object/context-mismatch task 24 h after the final seizure (Fig. 5a). No seizure controls showed preference for the novel object, indicating intact memory formation (Fig. 5b). In contrast, rats having previously undergone seizure activity that were treated with vehicle failed to remember the familiar object and scored significantly poorer than control rats (F_8,52_ = 5.64, p < 0.0001; Fig. 5b). Acetaminophen or nifedipine treatment which blocked repeated PIH also prevented the cognitive deficits (Fig. 5b) as indicated by the average discrimination index being very similar to that of control rats. Finally, we found that rats exposed to repeated seizures and tested again 2 weeks after kindling failed to remember the familiar object and scored significantly lower than controls (post hoc test p = 0.02; Fig. 5b). These findings cannot be attributed to differences in kindling epileptogenesis and provide evidence that repeated PIH episodes lead to long-term interictal cognitive deficits. Notably, the severity of PIH had a significant, negative, and linear relationship with performance on the task (Y =  − 8.306e−005 × X + 0.6411, R^2^ = 0.35, p = 0.0007), indicating a poorer performance of rats that had more severe hypoxia (Fig. 5c).

### Mitochondrial function is impaired interictally

**Figure 6:**
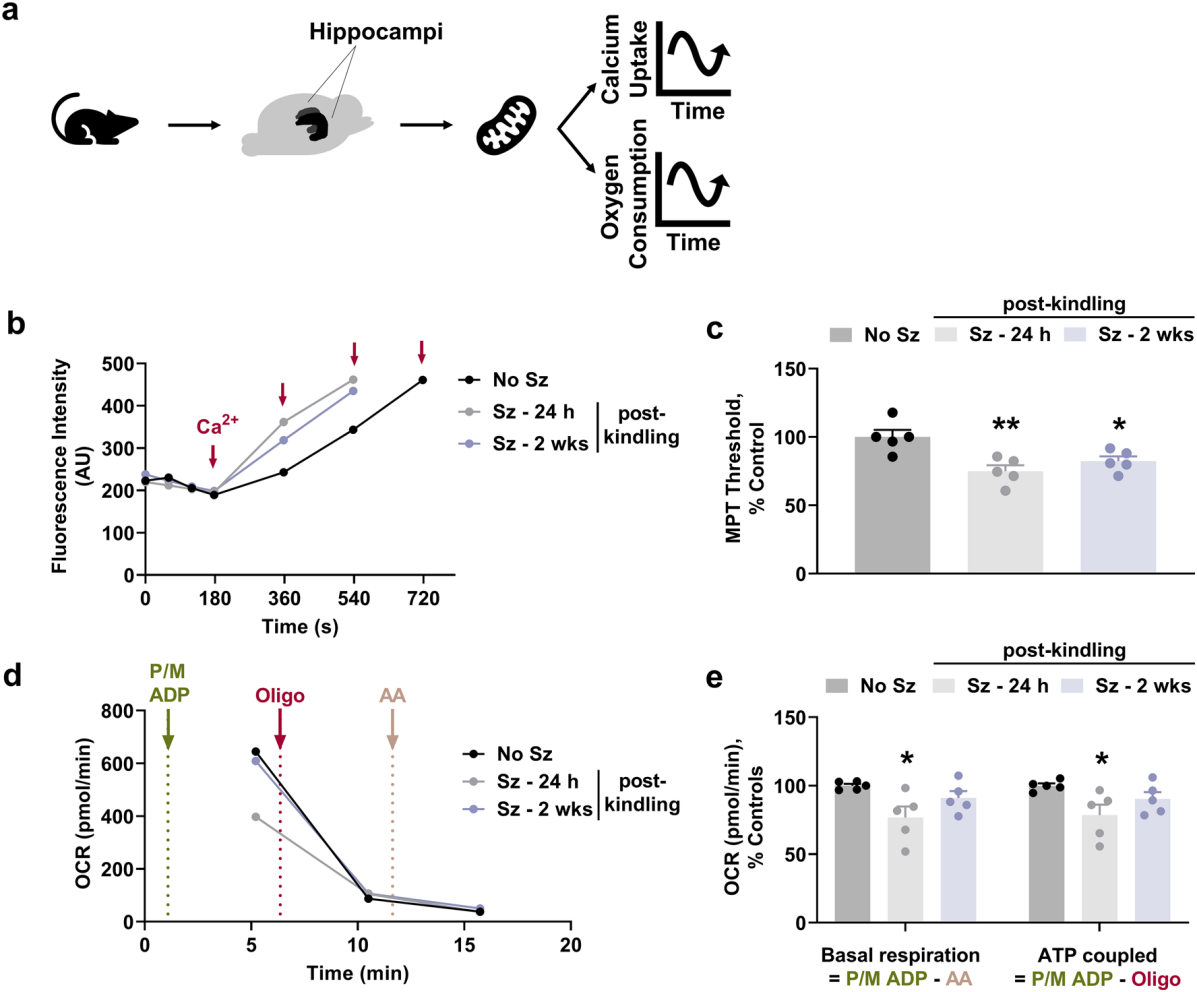
Mitochondrial function is impaired in between seizures and episodes of PIH. Acutely isolated mitochondria from both rat hippocampi were used to assess mitochondrial function. (**a**) Schematic representation of experimental design for the assessment of mitochondrial function. (**b**) Representative traces (n = 1 each group) depicting the capacity of mitochondria to sequester calcium. Red arrows represent calcium administration. (**c**) Quantification of mitochondrial permeability transition (MPT) threshold for the different experimental groups (n = 5) normalized to control rats. The Shapiro–Wilk test indicated data are normally distributed. Data are shown as mean ± SEM, followed by one-way ANOVA and Tukey’s post hoc test (*p < 0.05; **p < 0.01). (**d**) Representative traces (n = 1 each group) depicting oxygen consumption rates (OCRs; pmol/min) in the presence of chemical modulators of mitochondrial respiration. (**e**) Quantification of basal respiration and ATP linked respiration for the different experimental groups (n = 5) normalized to control rats. The Shapiro–Wilk test indicated data are normally distributed. Data are shown as mean ± SEM, followed by one-way ANOVA and Tukey’s post hoc test (*p < 0.05). *No Sz* no seizure, *Sz* seizure, *h* hours, *wks* weeks, *P/M* pyruvate/malate, *ADP* adenosine diphosphate, *Oligo* oligomycin, *AA* antimycin.

Recently, it has been observed that changes in mitochondrial function are sufficient to affect postictal hypoxia and hippocampal oxygenation during kindling^6^. We investigated how mitochondrial function changed at 24 h and 2 weeks after the last seizure by using mitochondria isolated from both rat hippocampi (Fig. 6a). We observed that mitochondria isolated from seizure exposed hypoxic rats retained significantly less calcium than mitochondria prepared from controls (F_2,12_ = 8.5, p = 0.005; Fig. 6b,c), indicative of a lower MPT threshold—a critical determinant of cell death. We further interrogated whether these changes are long-lasting. The impairment of calcium buffering capacity was persistent, lasting at least 2 weeks (Fig. 6b,c). In addition, we found significantly lower basal respiration in mitochondria isolated from seizure exposed hypoxic rats compared to control rats (F_2,12_ = 4.58, p = 0.03; Fig. 6d,e). Mitochondria taken from hypoxic rats having previously undergone seizure activity also exhibited lower ATP production (F_2,12_ = 3.95, p = 0.04; Fig. 6d,e). These changes were somewhat persistent but did recover to baseline over 2 weeks (Fig. 6d,e). These findings provide evidence that mitochondrial function is altered in between seizures and mitochondrial permeability becomes chronically compromised.

## Discussion

A plethora of seizure diseases including temporal lobe epilepsy are accompanied by severe interictal cognitive deficits. These deficits refer to impaired cognitive functioning of individuals that persist in between seizures^1^. It is estimated that approximately 40–60% of people with epilepsy have some form of interictal cognitive deficts^13^. Tasks that require attention, concentration, and learning may become arduous, hindering academic performance, occupational success, and overall quality of life. Despite being frequently observed, the precise mechanisms behind these cognitive deficits remain uncertain.

Our study provides evidence supporting the involvement of repeated episodes of postictal hypoxia (PIH) in the development of interictal cognitive deficits. Firstly, we determined that repeated kindled seizures in the hippocampus consistently leads to repeated PIH events, which progressively increase in severity over time. Notably, we observed a direct correlation between the severity of hypoxia and the magnitude of seizure-induced interictal memory deficits. By effectively preventing the occurrence of PIH events without interfering with kindling-induced epileptogenesis, we successfully preserved cognitive function during the period between seizures and prevented long-term brain dysfunction.

It is well-known that recurrent seizures can impair long-term potentiation (LTP), a critical process for learning and memory^14^. In line with this, previous studies have shown that PIH prevents the induction of LTP and rats exposed to hypoxia during the training phase of the novel object/context-mismatch task exhibit impairment in memory formation when tested 24 h later under non-hypoxic conditions^15^. In our current experiment rats were not hypoxic during training, but we still observed seizure-induced deficits in performance 3 days after their last seizure indicating that postictal and interictal manifestations may share similar underlying mechanisms. This emphasizes targeting PIH for therapeutic interventions.

In this study, we also found that recurrent seizures and PIH episodes lead to a permanent compromise in cellular calcium homeostasis, evidenced by increased mitochondrial membrane permeability. Calcium plays a crucial role in synaptic activity and memory formation. Previous research by Kim et al. indicates that cytosolic calcium alone is insufficient to trigger LTP without mitochondria involvement. Interfering with mitochondrial calcium uptake completely inhibits spinal LTP^16^. Therefore, the long-term disruption in mitochondria calcium buffering in our model may contribute to the development of interictal cognitive comorbidities.

A significant and positive correlation between cognitive alterations and seizure-induced oxidative damage has previously been reported^17, 18^. Targeting mitochondrial dysfunction was previously shown to be effective in reducing seizure-induced oxidative stress and prevent postictal cognitive dysfunction in kindled rats^6^. In this view, calcium has been demonstrated to be an endogenous modulator of reactive oxygen species (ROS) production, with mitochondrial calcium overload facilitating ROS formation^19–21^. In addition, we found that recurrent seizures and episodes of PIH lower mitochondrial metabolism as demonstrated by a decrease in basal oxygen consumption. Under low metabolic rates, the mitochondrial electron transport chain (ETC) remains in a chronically reduced state and this environment favours ROS production. Overwhelming levels of ROS, when not regulated, lead to oxidative stress. Notably, seizure induced-excessive oxygen conversion into reactive species has also been shown to contribute to postictal hypoxia^6^. It may, therefore, also explain the transient decrease in oxygen levels between seizures that we measured as well as their recovery in the absence of seizure and seizure-induced ROS. Furthermore, ROS affects cerebral vasculature—with high concentration of ROS acting as a cerebral vasoconstrictor and this may also explain the transient decrease in interictal oxygen levels in our model. It would be clinically relevant to determine if people with epilepsy have a chronically, but subtly hypoperfused seizure onset zones compared to seizure-free regions. We know that postictal hypoperfusion can be imaged with arterial spin labelling MRI and CT perfusion^22, 23^, which is concordant with the presumed seizure onset zone. However, it should be determined if this region is chronically hypoperfused interictally.

In summary, our data provide evidence that severe hypoxia triggered by seizures could have a significant impact on various aspects of epilepsy. Specifically, the cognitive impairments linked to temporal lobe epilepsy, which have been extensively studied but lacked precise mechanisms, appear to be related to the occurrence of postictal hypoxia. Our study also corroborates a mitochondrial underpinning of seizure-induced postictal hypoxia and its associated detrimental effects.

Seizure control is the main driver of epilepsy treatment. We treat people because they have seizures that have observable behavioural consequences. However, growing evidence indicates that PIH, and not the seizure per se, is responsible for several transient and chronic comorbidities. Focusing on seizures only as an electrical phenomenon ignores this other vascular and metabolic component that can cause cognitive difficulties and impair the function/performance of one third of people with epilepsy that do not respond to currently available anti-seizure drugs or where seizure control is poor. Treatments should be developed to suppress abnormal hypoxia that interfere with cognitive processes. We should treat both the seizures and the hypoxic attacks that follow.

## Methods

### Animals

Male Hooded Long-Evans rats (N = 81; 250–300 g at the time of surgery; Charles River, Canada) were housed individually in plastic cages with free access to food and water on a 12 h light/dark cycle (lights on at 7:00 AM) in a temperature (21 ± 3 °C) and humidity (60 ± 10%) controlled facility. In accordance with the Canadian Council for Animal Care guidelines, all experimental procedures were approved by the Health Sciences Animal Care Committee at the University of Calgary (AC16-0272, AC20-0170). All procedures complied with ARRIVE guidelines.

### Electrode and optode implantation

Rats were implanted under (5% induction and 1–2% maintenance) isoflurane anesthesia with a bipolar Teflon-insulated stainless-steel electrode 178 µm in diameter (A-M systems, Sequim, WA, USA) connected with gold amphenol male pins (CDM electronics, Turnersville, NJ, USA) and an oxygen-sensing probe (Oxford Optronix, Abingdon, UK). Rats were administered subcutaneously (SQ) with the antibiotic Baytril (10 mg/kg) and the analgesic buprenorphine (0.05 mg/kg) for analgesia. Lidocaine (15 mg/kg, SQ) was administered at the incision site as a local anesthetic. Burr-holes were drilled, and electrode and optode stereotaxically implanted into the ventral (from bregma, mm: AP − 5.0; ML 5.0; DV − 7.0) and dorsal (from bregma, mm: AP − 3.0; ML 3.5; DV − 3.5) right hippocampus, respectively. Stainless steel screws and dental cement were used to secure the implanted devices to the skull, with one of the four screws serving as a ground reference. After surgery, rats were administered buprenorphine (0.05 mg/kg, SQ) twice a day for 3 days. All rats were allowed at least one week of recovery prior to experimentation.

### Electrical kindling and oxygen recordings

Rats were unilaterally stimulated (60 Hz, 1 s train of 1 ms biphasic rectangular wave pulses) by using small incremental steps of current (50 µA; Grass S88 stimulator, Natus Neurology, Warwick, RI) in the ventral hippocampus to determine afterdischarge thresholds (ADTs; defined as the minimum amount of stimulus that evokes an electrographic seizure). Then, the rats were repeatedly stimulated once a day at an intensity of 100 µA above threshold to ensure that a seizure was elicited every time for 20 consecutive days—typical number of stimulations that lead to reliable postictal hypoxia profiles regardless of seizure severity^24^. Electrographic seizures without accompanying clinical manifestations were counted as seizures. For behavioral seizures, we assessed them using the five-stage seizure scale outlined by Racine^25^. No seizure controls were placed in the recording arena but did not receive kindling stimulation. An OxyLite Pro (Oxford Optronix, Abingdon, UK) was used to monitor real-time absolute measurement of dissolved oxygen in mmHg at 0.33 Hz (20 samples per min). On test days, rats were connected to the LFP and oxygen-sensing system and allowed at least 5 min of adjustment before any measurements were taken. Oxygen levels were recorded before, during, and after a seizure was elicited. The electrodes were disconnected once the LFP returned to baseline. Local hippocampal oxygen tension was recorded until oxygen return to baseline.

### Pharmacology

Pharmacological agents and dosages were established using insights from our initial study^5^, where various drugs were examined to identify potential candidates for averting postictal severe hypoxia. While the prevention of postictal hypoxia has been demonstrated through the administration of L-type calcium channel antagonist nifedipine either before or after seizures, only pre-seizure administration of the COX-inhibitor acetaminophen proves effective^5^. In our study, rats were administered with acetaminophen (250 mg/kg dissolved in 100% DMSO; intraperitoneally or i.p.; Sigma) or its vehicle 30 min before each kindling session for 20 days. Nifedipine (15 mg/kg dissolved in 100% DMSO; i.p.; Cayman Chemicals) or its vehicle was given to another cohort of rats immediately after each electrically evoked seizure for 20 consecutive days, enhancing the clinical relevance of our approach.

### Novel object/context-mismatch

Rats were tested in the novel object/context mismatch task, a form of hippocampal-dependent recognition memory^26, 27^. Rats were habituated to two different contexts for 10 min each, one immediately after the other, for two consecutive days. Context A was a large (60 cm × 60 cm) white box, housed in a well-lit room. Context B was a large, pentagonal black prism (60 cm in diameter), housed in a dimly lit room. On the third day (test day), rats were placed in each context contained a unique pair of identical objects for 5 min. Following a 5-min delay, rats were re-exposed to one of the contexts, this time with one object from each, and allowed to investigate the objects for 3 min. A discrimination index (time spent investigating the novel object/context pairing divided by total investigation time) was calculated. Values above 0.5 indicates intact preference for the novel object.

### Mitochondrial isolation and protein assay

Mitochondria were isolated using the Ficoll density centrifugation method. On test days, rats were euthanized with isoflurane overdose and immediately decapitated. The brains were rapidly removed, and the hippocampi were dissected. The tissue was submerged in ice-cold isolation buffer [215 mM mannitol, 75 mM sucrose, 0.1% BSA, 20 mM HEPES, and 1 mM EGTA at pH 7.2 adjusted with KOH] and then gently homogenized. Following a first step of centrifugation at 1300 RCF for 3 min at 4 °C, the supernatant was collected and centrifuged again at 13,000 RCF for 10 min at 4 °C. The pellet was resuspended in 300 µL isolation buffer and then placed in a nitrogen disruption bomb at 1200 psi for 10 min at 4 °C to release mitochondria from synaptosomes. Following pressure-release, the sample was collected and loaded on top of a Ficoll separation column consisting of a 10% Ficoll layer and a 7.5% Ficoll layer. The sample and the Ficoll column were then centrifuged at 32,000 rpm for 30 min at 4 °C. The resultant pellet was resuspended in 1 mL of isolation buffer without EGTA and centrifuged at 10,000 RCF for 10 min at 4 °C to further purify the mitochondrial sample. The final pellet was then resuspended in 20 µL isolation buffer without EGTA to yield a final concentration of approximately 10 mg/mL and stored on ice. Protein concentration was determined using the bicinchoninic acid (BCA) protein assay and measuring absorbance at 562 nm with a Synergy HT Plate Reader (BioTek, Winooski, VT, USA).

### Mitochondrial calcium buffering capacity and membrane permeability transition (MPT) threshold testing

Calcium Green 5N (CaG5N) fluorescent indicator was used to monitor extramitochondrial calcium and assess the ability of isolated mitochondria to buffer calcium and determine the calcium induced MPT threshold. Higher fluorescence is indicative of reduced calcium uptake and lower MPT threshold. Mitochondria were suspended in 2 mL respiration buffer. Mitochondria were then incubated in a constantly stirred cuvette at 37 °C with 100 nM CaG5N (excitation 506 nm, emission 532 nm), 5 mM pyruvate, 2.5 mM malate, 150 µM ADP in the spectrophotometer (Shimadzu RF-5301, Mandel, Guelph, Ontario, Canada). Scan began with a baseline measurement followed by the addition of 32 mM CaCl_2_ to the cuvette every 3 min until the mitochondria were no longer able to buffer the added calcium. The time-point at which the CaG5N signal was 150% above the baseline was the threshold for mitochondrial MPT^28^.

### Mitochondrial bioenergetics assay

A Seahorse Bioscience XFe24 extracellular Flux Analyzer was used to measure mitochondrial metabolism in isolated mitochondria. Prior to experimentation, 1.5 mL of XF calibrant solution (Agilent, Santa Clara, CA, USA) was added to each well of a 24-well assay cartridge (Agilent). The cartridge was incubated overnight at 37 °C without CO_2_. On test days, cartridge’s injector ports were loaded with mitochondrial substrates or inhibitors (port A: 5 mM pyruvate, 2.5 mM malate, and 4 mM adenosine diphosphate, (ADP); port B: 2 mg/mL oligomycin (Oligo) used to block ATP synthase; port C: 2 mM antimycin (AA) used to inhibit complex III of the mitochondrial electron transport chain. The cartridge was transferred to the Seahorse XFe24 Flux Analyzer to allow automatic calibration of optical sensors. Next, isolated mitochondria were diluted to 2.5 µg/50 µL in 37 °C respiration buffer [125 mM KCl, 2 mM MgCl_2_, 2.5 mM KH_2_PO_4_, 2 mM HEPES, 0.1% BSA at pH 7.2 adjusted with KOH]. Then, 50 µL of mitochondrial suspension was added to each well (except for background correction wells A1, B3, C4, and D6) of a 24-well cell culture microplate (Agilent). The plate was centrifuged at 2000 rpm for 20 min at 4 °C. After centrifugation, 450 µL of 37 °C respiration buffer was gently added to each well to reach a final volume of 500 µL per well. The plate was then placed into the calibrated Analyzer and real-time measurements of oxygen consumption rate (OCR) were performed. All the compounds were injected sequentially through the ports at pre-defined intervals to reveal key parameters of mitochondrial respiration: basal respiration calculated following AA injection and ATP linked respiration calculated following Oligo injection.

### Statistical analysis

Data analyses were carried out with GraphPad Prism 9 (GraphPad, La Jolla, CA, USA). Sample size was a priori determined based on approximations informed by pilot study data from our laboratory, power set at 0.8, and α = 0.05 using G*Power 3.1 (Institute for Experimental Psychology, Dusseldorf) as well as following the principles of the three Rs (Replacement, Reduction, and Refinement; https://www.nc3rs.org.uk/the-3rs). Further, the minimum number of animals required to derive statistically significant oxygen data was based on Farrell et al.^5^. Data were tested for normality using a Shapiro–Wilk test. Statistical significance was assessed using one-way analysis of variance (ANOVA) followed by Tukey’s test (or Sidak’s test when appropriate). Two-way ANOVAs followed by Tukey’s test (or Sidak’s test when appropriate) were used to compare more than two groups when more than one factor was investigated concurrently. Repeated measure statistics were used for all within subject experiments. For the groups that were not normally distributed, either Friedman or Kruskal–Wallis ANOVA tests were used. Linear regression was used to analyze the severity of PIH and performance on the behavioural task.

## Data Availability

The data underlying this article will be shared on reasonable request to the corresponding author.
